# Genitourinary Microbiome and Volatilome: A Pilot Study in Patients with Prostatic Adenocarcinoma Submitted to Radical Prostatectomy

**DOI:** 10.3390/cancers17233841

**Published:** 2025-11-29

**Authors:** Layla Musleh, Sara Passerini, Francesca Brunetti, Linda Maurizi, Giulio Bevilacqua, Lorenzo Santodirocco, Beatrice Sciarra, Martina Moriconi, Caterina Fraschetti, Antonello Filippi, Maria Pia Conte, Valeria Pietropaolo, Marisa Di Pietro, Simone Filardo, Alessandro Sciarra, Catia Longhi

**Affiliations:** 1 Department of Public Health and Infectious Diseases, Microbiology Section “Sapienza” University of Rome, P. le Aldo Moro 5, 00185 Rome, Italy; layla.musleh@uniroma1.it (L.M.); sara.passerini@uniroma1.it (S.P.); francesca.brunetti@uniroma1.it (F.B.); mariapia.conte@uniroma1.it (M.P.C.); valeria.pietropaolo@uniroma1.it (V.P.); marisa.dipietro@uniroma1.it (M.D.P.); simone.filardo@uniroma1.it (S.F.); catia.longhi@uniroma1.it (C.L.); 2 Department of Pediatric Surgery, San Camillo-Forlanini Hospital, Circonvallazione Gianicolense 87, 00152 Rome, Italy; 3 Department of Maternal-Infant and Urologic Sciences, “Sapienza” University of Rome, Viale Policlinico 155, 00161 Rome, Italy; giulio.bevilacqua@uniroma1.it (G.B.); lorenzo.santodirocco@uniroma1.it (L.S.); martina.moriconi@uniroma1.it (M.M.); alessandro.sciarra@uniroma1.it (A.S.); 4 Department of Chemistry and Technologies of Drug, “Sapienza” University of Rome, P.le Aldo Moro 5, 00185 Rome, Italy; beatrice.sciarra@uniroma1.it (B.S.); caterina.fraschetti@uniroma1.it (C.F.); antonello.filippi@uniroma1.it (A.F.)

**Keywords:** prostate cancer, genitourinary microbiome, volatile metabolites, sexually transmitted pathogens, HPyVs

## Abstract

**Background/Objectives**: The genitourinary microbiome and metabolome may contribute to prostate cancer (PC) biology, but evidence remains limited. This pilot study characterizes the urinary microbiota and volatilome in men with PC and investigates microbial and viral DNA in prostate tissue, comparing findings with benign prostatic hyperplasia (BPH). **Methods**: We prospectively enrolled 21 non-metastatic PC patients undergoing radical prostatectomy and 17 BPH controls. Lesional and non-lesional prostate tissues and urine were collected from PC patients, as well as urine samples from BPH participants. DNA samples were tested for sexually transmitted pathogens by multiplex real-time PCR. Urine and prostate tissue were analyzed for human polyomaviruses (JCPyV, BKPyV, MCPyV) by qPCR, bacterial profiles via 16S rRNA gene sequencing, and urinary volatile organic metabolites (VOMs) using HS-SPME/GC-MS. Microbial and metabolic profiles were compared, and taxa–metabolites were assessed. **Results**: JCPyV and BKPyV were detected in urine and tissue from PC patients; MCPyV was detected only in tissue, at low frequency. In BPH, viral prevalence was lower and MCPyV was absent. JCPyV/BKPyV co-infection was common in cancer. No sexually transmitted pathogen emerged. PC patients showed greater urinary microbial diversity and five enriched genera, along with specific metabolic pathways. 36 urinary VOMs were identified, with 14 differing significantly, with positive correlations between PC-associated genera and metabolites. In contrast, prostate tissue was low-biomass, dominated by *Pseudomonas*, and showed no significant differences between lesional and non-lesional areas. **Conclusions**: This preliminary, hypothesis-generating study indicates that urinary, rather than tissue, microbial and volatilome signatures show clearer differences between PC and BPH. These findings suggest possible microbiota–metabolite interactions in PC but require validation in larger cohorts.

## Simple Summary

Prostate cancer may be influenced not only by genetics and hormones but also by the microorganisms and metabolic substances present in the prostate and in urine. Understanding how these elements interact could help researchers better describe the biological environment associated with this disease. In this study, we examined prostate tissue and urine from men with prostate cancer and compared them with samples from men with non-cancerous prostate enlargement. We looked for viruses, analyzed bacterial communities, and studied the volatile molecules released in urine. While prostate tissue showed no major differences between diseased and non-diseased areas, urine samples from patients displayed greater microbial diversity and distinct metabolic patterns. These results offer early insights into how microbes and metabolism may be linked to prostate cancer and provide a basis for future research.

## 1. Introduction

Prostate cancer (PC) is the second most common malignancy in men and the third leading cause of cancer-related death worldwide [1,2]. It is a multifactorial disease, influenced by genetic, environmental, and immunological determinants [3,4]. Established risk factors include age, family history, ethnicity, diet, and microbial infections [3,4,5,6,7,8,9]. Among infectious agents, bacteria, including *Cutibacterium (Propionibacterium) acnes*, *Neisseria gonorrhoeae*, *Escherichia coli*, and *Mycoplasma* spp., have been implicated in prostate carcinogenesis, although evidence remains limited [8,9]. A possible viral contribution has also been investigated [10]. *Human Papillomavirus* (HPV), *Epstein–Barr virus* (EBV), several human herpesviruses (HHVs), such as *Cytomegalovirus* (CMV) and *Kaposi’s sarcoma-associated herpesvirus* (KSHV), have been detected in prostate tumor tissue [11,12,13]. Human Polyomaviruses (HPyVs) have also been investigated in prostate carcinogenesis [14,15]. Among them, only Merkel cell polyomavirus (MCPyV) has a proven oncogenic role in humans (Merkel cell carcinoma) [16]. *JC* and *BK* polyomavirus (JCPyV and BKPyV) display oncogenic properties [17], and their DNA or proteins have been detected in both malignant and benign prostate tissues [15,18]. In particular, JCPyV has been associated with higher prostate-specific antigen (PSA) and Gleason scores [19,20,21,22], but evidence remains inconsistent. Several studies reported no significant differences in prevalence between cancerous and benign prostates, or no association with BKPyV [19]. MCPyV DNA has occasionally been identified in prostate tumors [19,23,24]. Recent attention has focused on microbial dysbiosis as a potential driver of cancer onset and progression [25,26,27], though findings remain inconsistent [19]. Beyond taxonomic composition, microbial metabolic output may be critical, as distinct communities can produce convergent metabolic profiles [28]. Notably, altered levels of citrate, leucine, valine, and taurine have been detected in both prostate tissue and urine, indicating potential for non-invasive metabolic biomarkers [29]. Yet, validation and standardization challenges persist [30,31]. Further exploratory work integrating microbial and metabolic analyses may help clarify these interactions [32,33]. Accordingly, this exploratory pilot study aims to characterize the microbial signatures of prostate tissue and urine and to delineate the urinary volatilome profile of men with histologically confirmed PC, comparing these findings with those of patients with benign prostatic hyperplasia (BPH). By integrating viral, bacterial, and metabolomic assessments across tissue and urine, the study sought to provide a comprehensive overview of the genitourinary microenvironment in PC.

## 2. Materials and Methods

### 2.1. Study Design

This prospective, single-center study was conducted at “Sapienza” University—Policlinico Umberto I in accordance with the Declaration of Helsinki after receiving ethics approval (Protocol No. 0309/2023, Approval No. 7084), and enrollment took place from January 2023 to January 2024.

### 2.2. Patient Selection Criteria

Cases included men of any age or ethnicity undergoing radical prostatectomy (RP) for a non-metastatic prostate adenocarcinoma. Exclusion criteria included any previous or current oncologic treatment or history, inflammatory or infectious conditions, hormonal/steroid therapy, recent antibiotics, or microbiota-modifying drugs. Controls were represented by men affected by benign prostatic hyperplasia (BPH). No formal sample size calculation was performed. This investigation was conceived as an exploratory, pilot study, and the sample size was determined by feasibility and by the number of eligible patients available during the recruitment period.

### 2.3. Clinical Procedures and Follow-Up

All patients underwent serum PSA testing and multiparametric magnetic resonance imaging (mpMRI). When PC was suspected, a targeted MRI/ultrasound fusion-guided biopsy was performed. Tumor risk was stratified according to European Association of Urology (EAU) guidelines and, bone scintigraphy or positron emission tomography-computed tomography (PET-CT) were performed in high-risk cases. Robotic-assisted laparoscopic (RP) followed multidisciplinary discussion. Tumors were histologically graded per the International Society of Urological Pathology (ISUP) system [34]. Postoperative monitoring included PSA every three months for two years and biochemical recurrence was defined as PSA > 0.2 ng/mL. Prostate-Specific Membrane Antigen (PSMA) PET-CT was used to assess recurrence or progression.

### 2.4. Specimen Collection

After overnight fasting, 100 mL of urine was collected by catheterization. Following prostatectomy, two tissue samples (~1 g each) were obtained: one from the lesional area and one from a macroscopically tumor-free zone (non-lesional), both confirmed by histology. In controls, urine was collected using the same procedure. All specimens were refrigerated at 4 °C, transported within 30 min, and stored at −20 °C until processing.

### 2.5. DNA Extraction from Urine and Prostate Tissue

DNA was extracted from urine (500 µL) and tissue using Quick-DNA MiniPrep Kit and Quick-DNA FFPE Kit (Zymo Research, Irvine, CA, USA), respectively, following the manufacturer’s instructions. The final elution volume was 200 µL DNA yield and purity were measured with a Synergy HT Take3 Microplate Reader (BioTek, Winooski, VT, USA).

### 2.6. Detection and Quantification of HPyVs DNA

Quantitative polymerase chain reaction (qPCR) was used to assess the prevalence and viral load of JCPyV, BKPyV, and MCPyV, targeting Large T Antigen (LTAg), Viral Protein 1 (VP1), and small T antigen (sTAg), respectively [35,36]. Viral loads (copies/mL) were derived from standard curves of ten-fold serial dilutions (10^8^–10^1^ copies/mL) of full-length genome plasmids. Reactions were run in triplicate with positive and negative controls.

### 2.7. Sexually Transmitted Pathogen Detection

DNA samples were tested for *Chlamydia trachomatis*, *Neisseria gonorrhoeae*, *Trichomonas vaginalis*, *Mycoplasma genitalium*, *Mycoplasma hominis*, *Ureaplasma urealyticum*, and *Ureaplasma parvum*, using the multiplex real-time PCR assay Anyplex II STI-7 Detection Kit (Seegene, Seoul, Republic of Korea) according to the manufacturer’s instructions. Each run included positive/negative extraction controls, and a no-template control (ultrapure PCR-grade water). Samples were analyzed in triplicate.

### 2.8. 16S rRNA Gene Sequencing, Processing, and Metagenomic Analysis

The V3–V4 regions of the 16S rRNA gene were PCR-amplified and sequenced via Illumina MiSeq (2 × 300 bp). Raw reads were merged using Usearch v.11 [37], primer-stripped via Cutadapt v.4 [38], and quality filtered via Trimmomatic v. 0.39 [39], setting “*SLIDINGWINDOW:5:30, MINLEN:50*”.

Reads were imported into QIIME2 [40] v2022.2 and denoised via DADA2 [41]. Reads were clustered into OTU97 using an *open reference approach*, using the Greengenes v13_8 database. OTUs found in <4 samples, having <50 reads, and representing <0.05% in each group were discarded.

Observed features, Chao1, Shannon, Faith’s PD indexes were computed for the α-diversity, while Bray–Curtis and Weighted UniFrac distances for the β-diversity. Taxonomic classification was performed with a Naive Bayes classifier, built upon the Greengenes rDNA v13_8 database. Functional inference was performed using PICRUSt2 [42].

### 2.9. Urine Metabolomics HS-SPME/GC-MS Analysis

2 mL of urine supernatant (centrifuged at 4000 rpm, 4 °C, 20 min) was placed in 6 mL vials, added with NaCl (0.57 g), acidified to pH 2 with 7 µL of HCl, and equilibrated for 30 min at 50 °C under continuous stirring at 200 rpm [43]. A DVB-PDMS fiber (Merck Life Science S.R.L.) was exposed in the headspace for 45 min at 50 °C to extract the volatile organic metabolites (VOMs) fraction of the sample to be introduced into the gas chromatograph (GC) inlet for 0.5 min. All the analyses were performed in triplicate using an Agilent Technologies 6850 GC combined with an Agilent Technologies 5975 mass spectrometer (Santa Clara, CA, USA). The following chromatographic conditions were employed: capillary column, HP-5MS (30 m × 0.25 mm inner diameter, film thickness 0.25 μm); inlet temperature, 250 °C; injection mode, splitless (valve opening after 0.2 min, split ratio 10/1); carrier gas, helium (99.995% purity) with a 1.0 mL/min flow; temperature programming, oven was kept at 40 °C for 5 min, then increased by 5 °C/min up to 200 °C, and maintained at this final temperature for 30 min. The mass spectrometer operating values were set as follows: EI energy, 70 eV; source and quadrupole temperatures, 230 °C and 150 °C, respectively; mass scan range, 50–350 *m*/*z*.

### 2.10. Data Pre-Processing

Data was converted to mzData (Chemstation software, E.02.00 version, Agilent Technologies) and processed using XCMS-online platform [44] to correct and align the retention times of the chromatograms. All the generated peak groups were normalized, obtaining the metabolite area expressed as percentage abundances for statistical testing.

### 2.11. R-Based Statistical Analysis

Statistical analyses were performed using R v.4.1.2. Friedman’s test (>2 dependent groups), followed by Wilcoxon rank sum test, and Mann–Whitney U-test (2 independent groups) were used to compare continuous variables. Permanova (1000 permutations) was calculated on β-diversity distance matrices. Differential abundance analysis (DAA) of taxa and functional pathways was carried out using Aldex2 [45]. Correlations between genera and metabolites were computed using the Kendall coefficient. When necessary, *p*-values were adjusted using the Benjamini–Hochberg FDR correction. *p*-values ≤ 0.05 were considered statistically significant.

## 3. Results

### 3.1. Participant Characteristics

Clinical and pathological features of the 21 PC patients and 17 BPH controls are summarized in Table 1. The groups were comparable in terms of age, BMI, and comorbidities, though prostate volume was significantly smaller and PSA levels significantly higher in the PC group. Most PC patients were classified as intermediate risk per EAU criteria, with tumors mainly staged as pT2 or pT3a and graded as ISUP 2. Following RP, positive surgical margins were observed in one-third of cases. During follow-up, three patients (14.3%) developed biochemical progression, while the remainder maintained undetectable PSA levels.

### 3.2. Detection of HPyVs in Urine and Prostatic Tissue from PC Patients

Among 21 PC patients, 13 (61.9%) were positive for JCPyV, 13 (61.9%) for BKPyV, and 2 (9.5%) for MCPyV. JCPyV and BKPyV were mainly detected in urine (61.9% and 57.1%), with lower prevalence in tissues: JCPyV in 19% non-lesional and 9.5% lesional samples, and BKPyV in 33.3% and 9.5%, respectively. JCPyV was significantly more prevalent in urine than in both non-lesional (*p* < 0.05) and lesional (*p* ≤ 0.001) tissues, whereas BKPyV differed only between urine and lesional samples (*p* < 0.05). qPCR confirmed this trend: JCPyV median viral load was 8.7 × 10^6^ copies/mL in urine vs. 1.38 × 10^3^ and 4.65 × 10^2^ copies/mL in non-lesional and lesional areas; BKPyV 1.33 × 10^5^ copies/mL in urine vs. 1.2 × 10^2^ and 8.5 × 10 in non-lesional and lesional samples. Both viruses showed significantly higher loads in urine than tissues (*p* < 0.05), with no difference between lesional and non-lesional areas (*p* > 0.05). MCPyV was undetectable in urine and detected at low copy numbers in 1 non-lesional (4.8%) and 2 lesional (9.5%) samples (Table 2).

### 3.3. Detection of HPyVs in Urine from BPH Controls

Among 17 urine samples from controls, 4 (23.5%) were positive for JCPyV (median 2 × 10^6^ copies/mL; CI 95% 2.1 × 10^4^–7.5 × 10^6^) and 4 (23.5%) for BKPyV (median 4.8 × 10^6^ copies/mL; CI 1.3 × 10^4^–9.2 × 10^5^). No significant differences in viral load were observed between PC and BPH patients (*p* > 0.05). MCPyV was not detected in any BPH sample.

### 3.4. HPyVs Coinfection Patterns in PC Patients

Several viral coinfection patterns were identified. JCPyV and BKPyV were the most frequent, co-detected in 10 patients (47.6%), while BKPyV and MCPyV coinfection occurred in only 2 (9.5%). No triple infections were detected. In addition, 2 patients (9.5%) showed JCPyV and BKPyV coinfection across multiple anatomical sites (urine, non-lesional and/or lesional tissues).

### 3.5. Detection of Sexually Transmitted Pathogens in Urine and Prostate Tissue

All urine and prostate tissue samples tested negative for *C. trachomatis*, *N. gonorrhoeae*, *T. vaginalis*, *M. genitalium*, *M. hominis*, *U. urealyticum*, and *U. parvum*.

### 3.6. Sequencing and Bioinformatics Analysis

Of the 80 metagenomic samples, only 78 were eligible for analysis. These included 20 lesional and 21 non-lesional prostatic samples, 21 catheterized urine samples from patients, and 16 urine samples from BPH controls. A total of 9,474,652 sequences were generated (median: 107,346.5; IQR: 68,462–258,580), which were reduced to 6,663,561 (median: 66,879.5; IQR: 38,875.7–127,262.7) after the initial filtering steps and resulting in 359 OTUs across the prostatic (OTUs = 353) and urine microbiome (OTUs = 351).

### 3.7. Comparisons Between Lesional and Non-Lesional Prostate Tissue Microbiota

**Figure 1:**
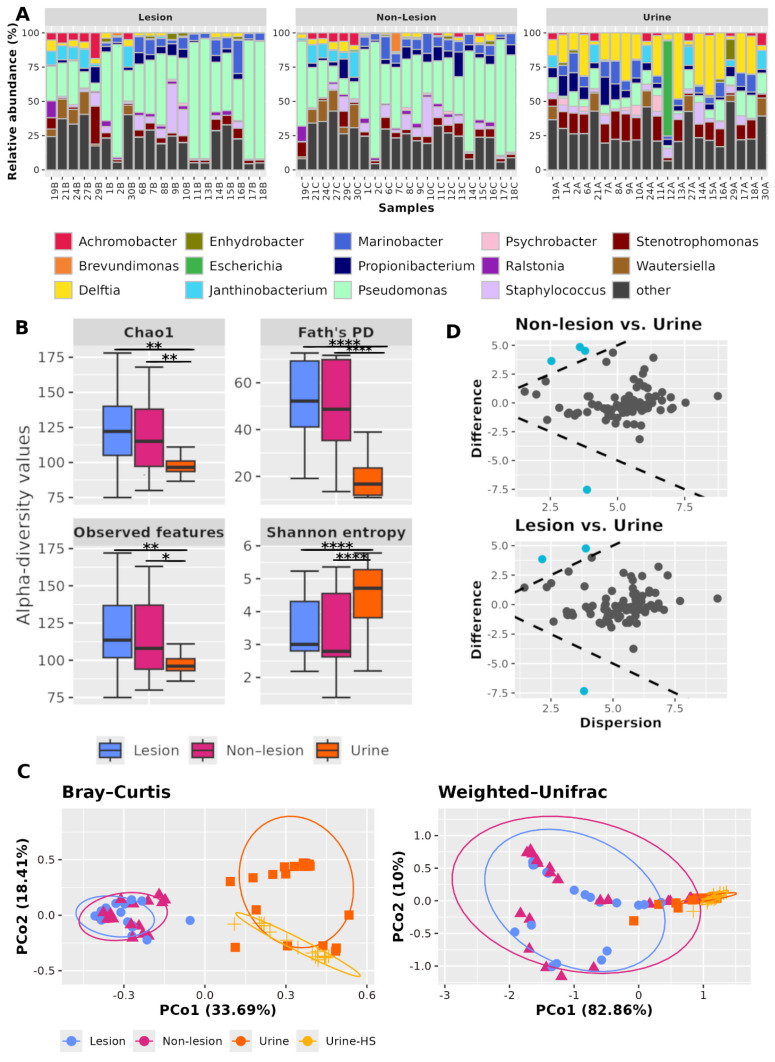
Comparisons between prostate and urine microbiota. (**A**) Microbial composition of prostate and urine microbiome in patients. Genus accounting for <10% in all groups was aggregated and labeled as “other”. (**B**) α-diversity boxplots, where asterisks indicate *p* significance. (**C**) β-diversity PCoA, where each sample for this study corresponds to a single dot. The percentage of total variance explained by the principal coordinates is reported on both axes. (**D**) DAA effect plot. Each dot represents one of the 85 bacterial genera, displaying on the x-axis its median difference in pairwise comparisons between groups (difference) and on the y-axis the maximum within-group variance (dispersion). Difference > 0 means that the genus is more abundant in urine samples than in the prostate. Blue dots represent the differentially enriched genera (*p* < 0.05 and ES > |1|). Notation: *lesion: prostate sample from lesional areas; non-lesion: prostate sample from non-lesional areas; Urine: urine sample from patients; Urine-HS: urine sample from BPH subjects*; *: *p* < 0.05; **: *p* < 0.01; ****: *p* < 0.0001.

First, we compared the prostate microbiota between the lesional and non-lesional areas. Although no statistically significant differences in composition were found between the two areas, a total of 80 distinct genera were detected, with 78 in the non-lesional area and 75 in the lesional area. As shown in Figure 1A, the microbial composition of lesional and non-lesional samples is very similar, with *Pseudomonas* representing the most abundant genus in both groups. Furthermore, *Pseudomonas* and *Staphylococcus* are the only genera found in all prostatic samples.

### 3.8. Comparisons of Microbiota in Prostate Tissue and Catheterized Urine

Urine and prostate tissue are anatomically and functionally connected, but whether urine can reflect the prostate tissue microbiome remains to be resolved. In our study, significant intra-individual differences were detected between prostate and urine samples. Compared to the prostate, α-diversity analysis revealed that the urine microbiome was characterized by lower richness (Figure 1B), a more even distribution of microbial abundances, and a community composed of more closely related microorganisms. Regarding β-diversity, statistically significant differences in composition were found between the prostatic and urine microbiomes, both in terms of microbial abundance (Bray–Curtis distance, plesion vs. urine and p_nonlesion vs. urine_: 0.0015) and phylogenetic relationships (Weighted Unifrac distance, p_lesion vs. urine_ and p_nonlesion vs. urine_: 0.0015) (Figure 1C).

A total of 78 genera were found in the urine microbiome (Figure 1A). DAA evidenced that *Ralstonia* was the only genus significantly more enriched in the prostatic samples (p_lesion vs. urine_: 0.002, ES: −1.77; p_nonlesion vs. urine_: 0.001, ES: −1.87), while Delftia (p_lesion vs. urine_: 0.0003, ES: 1.65; p_nonlesion vs. urine_: 0.0003, ES: 1.40) and Sphingobium (p_lesion vs. urine_: 0.0008, ES: 1.06; p_nonlesion vs. urine_: 0.0001, ES: 1.24) were significantly more depleted. Furthermore, *Sediminibacterium* was found to be significantly more enriched in the non-lesional prostatic microbiome than in urine (*p* = 0.003, ES: 1.16) (Figure 1D).

### 3.9. Comparisons of Urine Microbiota in PC and BPH Patients

**Figure 2:**
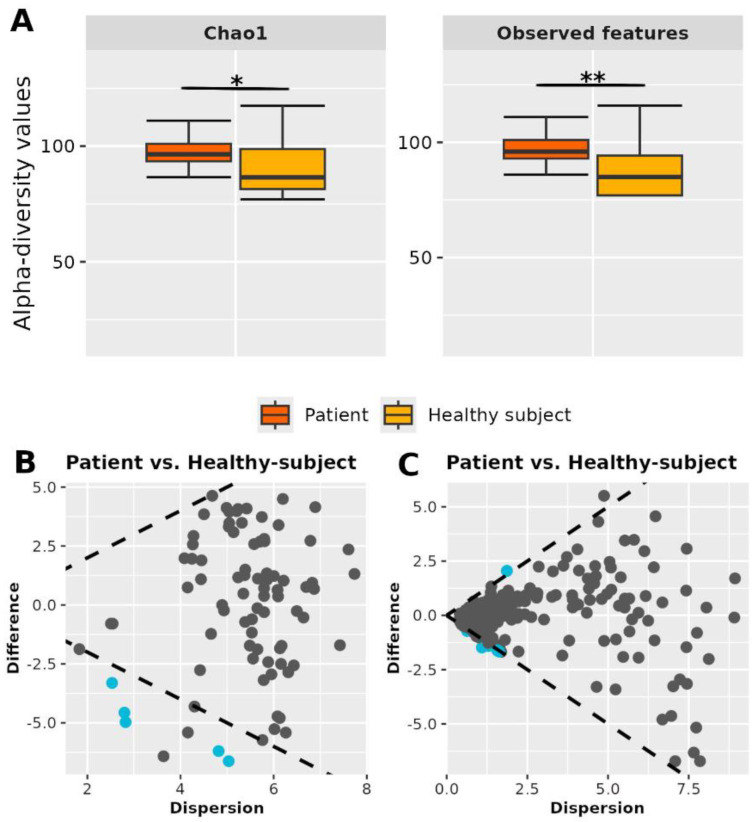
Urine sample comparisons between patients and healthy subjects. (**A**) α-diversity boxplot where the asterisk denotes *p*-value significance. (**B**,**C**) Effect plot representing the DAA (**B**) and functional analysis (**C**) results. Each dot represents a single genus or pathway, displaying on the x-axis its median difference in pairwise comparisons between groups (difference) and on the y-axis the maximum within-group variance (dispersion). Difference > 0 means that the genus or the pathway is more enriched in patients. Blue dots represent the differentially enriched genera or pathways (*p* < 0.05 and ES > |1|). Notation: *: *p* < 0.05; **: *p* < 0.01.

Patients exhibited significantly higher richness in their urine microbiota (Figure 2A). Additionally, statistically significant differences in microbial composition were found between the groups, based on both microbial abundance (Bray–Curtis distance, *p* < 0.001) and phylogenetic relationships (Weighted UniFrac distance, *p* < 0.001) (Figure 1B). The urine microbiome of both patients and healthy individuals comprised 85 genera, 72 of which were found in healthy subjects. DAA identified 5 of these bacterial genera being significantly more enriched in patients, namely *Delftia* (*p* < 0.0001, ES: −1.34), *Sphingobium* (*p* < 0.0001, ES: −1.70), *Stenotrophomonas* (*p* < 0.0001, ES: −1.70), *Sphingopyxis* (*p* = 0.0004, ES: −1.17), and *Parvibaculum* (*p* = 0.001, ES: −1.18) (Figure 2B). Assessment of the functional potential of the urine microbiome revealed the presence of 6 pathways significantly associated with patients, namely *ectoine biosynthesis* (*p* < 0.0001, ES: −1.33), *4-aminobutanoate degradation* V (*p* = 0.0006, ES: −1.07), *octane oxidation* (*p* = 0.0003, ES: −1.07), *vanillin and vanillate degradation* I (*p* = 0.0009, ES: −1.04), *superpathway of vanillin and vanillate degradation* (*p* = 0.0008, ES: −1.04) and *vanillin and vanillate degradation* II (*p* = 0.0008, ES: −1.01), while *Bifidobacterium* shunt (*p* = 0.0003, ES: 1) and heterolactic fermentation (*p* = 0.0002, ES: −1.02) pathways were significantly associated with healthy subjects (Figure 2C).

### 3.10. HS-SPME/GC-MS Analysis of the Urine VOM Fraction

**Figure 3:**
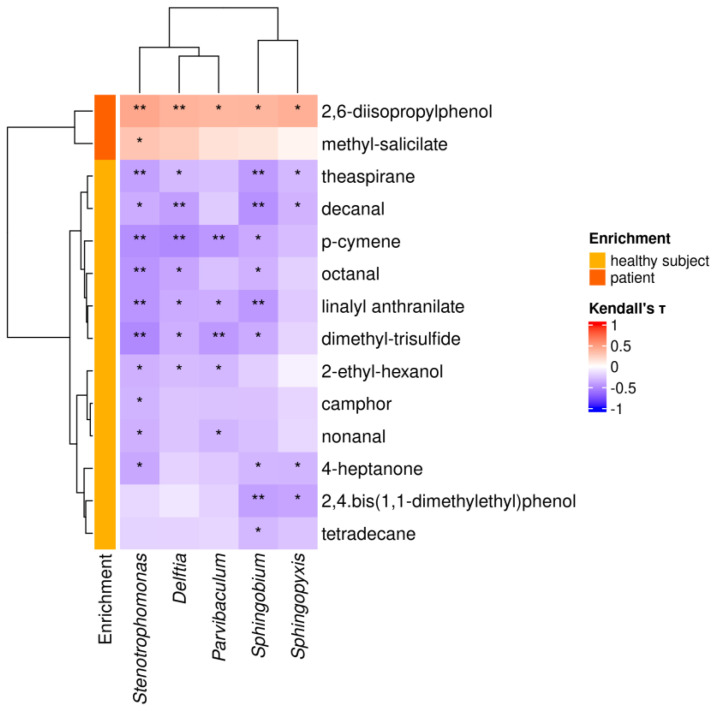
Genus–metabolite correlation analysis. Heatmap reporting correlations between the abundance of differentially enriched genera and metabolites in urine. Asterisks indicate statistically significant correlations. Dendrograms were generated using the Ward’s D2 hierarchical clustering method. Notation: *: *p* < 0.05; **: *p* < 0.01.

HS-SPME/GC-MS was employed to extract and assess potential biomarkers of PC within the VOM fraction of the collected urine samples. A total of 36 volatile compounds were detected, of which 14 remained differentially enriched after removing column-bleeding peak areas (Table 3). As the enrichment (or depletion) of particular bacterial genera and metabolites turn out to be significantly associated with the urine of PC patients, we sought to evaluate whether the variation in abundance of these two components might be correlated. Statistically significant correlations showing a weak strength (∣τ∣ ∈ [0.3,0.5]) were found between the 5 PC-associated genera and 14 of the differentially enriched metabolites (Figure 3).

Among the PC-associated metabolites, we observed significant positive correlations between *2,6-diisopropylphenol* and all 5 PC-associated genera (τ ∈ [0.37,0.45]), while *methyl-salicylate* showed a significant positive correlation only with *Stenotrophomonas* (τ = 0.30). Negative correlations were found with the 12 PC-depleted metabolites, where the highest number of significant correlations were found with *Stenotrophomonas* (τ ∈ [−0.51,−0.32]), *Sphingobium* (τ ∈ [−0.47,−0.30]) and *Delftia* genera (τ ∈ [−0.50,−0.30]).

## 4. Discussion

This study integrated viral, bacterial, and metabolomic profiling of prostate tissue and urine to investigate the genitourinary microenvironment in PC. We initially hypothesized that microbial alterations within prostatic tissue could differ between lesional and non-lesional areas, as suggested in previous reports. However, our findings did not reveal significant differences between malignant and non-malignant regions, with all samples showing a low-biomass microbial profile dominated by *Pseudomonas*. These observations do not support a major intraprostatic dysbiosis in our cohort and reflect the variability reported across previous tissue-based analyses. Cavarretta et al. showed minimal differences among tumor and adjacent regions [46]; Feng et al. identified no α-diversity changes, with a core microbiome featuring *Pseudomonas*, *Escherichia*, *Acinetobacter*, and *Propionibacterium* spp., and suggested possible negative correlations between *Pseudomonas* abundance and metastatic progression [47]. In line with this, Gonçalves et al. observed higher *Pseudomonas* levels in non-cancerous tissues and linked *Cutibacterium* and *Staphylococcus* to aggressiveness [48,49]. Li et al. later described *Pseudomonas* enrichment in BPH, suggesting a role in progression through NF-κB activation by lipopolysaccharide [50].

Taken together, these negative findings argue against a major tissue-level dysbiosis in our cohort. In addition, the low-biomass nature of prostate tissue increases the risk of environmental or reagent-derived contamination, and genera such as *Pseudomonas* are known reagent contaminants [51]. For these reasons, we interpreted tissue-derived microbial signals with caution.

In light of the absence of clear tissue-level distinctions in our dataset, we focused subsequent analyses on urinary samples, which exhibited clearer group-level differences and higher microbial richness. Urine, therefore, provided a more informative matrix for detecting both microbial and metabolomic alterations associated with PC, allowing a more meaningful interpretation of potential microbiota–metabolite interactions. The hypothesis that the urinary tract may serve as a primary route for microbial colonization of the prostate led to a comparison between prostatic tissue and catheterized urine microbiota from the same individuals [50,52]. α-diversity analysis indicated lower richness and a more even abundance distribution in the urinary microbiome, whereas significant differences in microbial composition and phylogenetic structure were observed in β-diversity analysis. *Ralstonia* was significantly more abundant in prostate samples, in contrast to *Delftia* and *Sphingobium* found more in urine. These patterns parallel prior findings from Okada and Li et al., who reported clear distinctions between urinary and prostatic microbiota in patients with BPH [50,53]. Our detection of *Ralstonia* corroborates Yow et al., who identified this genus in high-grade PC [54], and aligns with reports of *Ralstonia pickettii* prevalence in tumor tissue [55].

In the urinary microbiome, 85 genera were identified with five—*Sphingobium*, *Stenotrophomonas*, *Sphingopyxis*, *Parvibaculum*, and *Delftia*—showing increased abundance in PC patients. Gonçalves’s study linked urinary dysbiosis to PC, potentially fostering chronic prostatic inflammation [48]. Urine, being metabolically rich, contains both host and microbial by-products. Shifts in gut microbiota may influence PC through a proposed “gut–prostate axis” [56]. Among enriched taxa, members of the *Sphingomonadaceae* family—such as *Sphingobium* and *Sphingopyxis*—are recognized for thriving in contaminated environments and participating in heavy-metal phytoremediation [57]. Heavy metal exposure has been implicated in prostate disease pathogenesis and shown to alter gut microbiota, whereas probiotics may counteract such dysbiosis [58,59].

While VOM profiles can reflect metabolic activity of both microbial and host origin, their interpretation in small exploratory cohorts should remain cautious [29,60,61,62,63,64]. Findings in this cohort revealed reduced aldehydes in PC patients, alongside a noted correlation between increased aldehyde dehydrogenase activity and tumor progression [65,66]. Other compounds like nonanal and octanal, generated through host or bacterial metabolism or the auto-oxidation of unsaturated fatty acids [67], have been recognized as non-specific inflammatory mediators [68] with pro-inflammatory effects [68,69]. In the literature, *Stenotrophomonas maltophilia* has been associated with aggressive PC features such as including Gleason score, TNM stage, PSA levels, and androgen receptor expression [70]. In our cohort, however, correlations between this genus and individual metabolites were weak (τ ≈ 0.3–0.5) and should therefore be considered preliminary and hypothesis-generating rather than clinically meaningful. Additionally, urinary 4-heptanone levels were reduced in PC patients, potentially stemming from the decarboxylation of plasticizer-related metabolites [71,72]. Similarly, decreased levels of 2-ethyl-1-hexanol may reflect metabolic alterations but cannot be interpreted as causally linked to tumor biology [73]. P-cymene, a monoterpene found in various foods [61,74] processed by cytochrome P450 enzymes, whose dysregulation in PC could impact its urinary excretion [75], showed lower urinary levels in PC patients, aligning with previously documented research [61]. Although p-cymene displays anti-invasive effects in vitro [76,77], such experimental data cannot be extrapolated to our clinical cohort. In BPH subjects, p-cymene has shown negative correlations with *Stenotrophomonas*, *Parvibaculum*, and *Delftia*. *Delftia*, enriched in the urine of PC patients in our study cohort, has been linked to cervical intraepithelial neoplasia [78]. *D. acidovorans* in PC tissue correlated with regulatory T-cell infiltration and downregulation of immune-related genes including LPCAT2, TL3, and TGFB2. These cross-study associations suggest possible immune–microbiota interactions, though the mechanistic relevance for PC remains uncertain [79].

The research also proposed that p-cymene may inhibit tumor development by enhancing the composition of intestinal flora, thus fostering the growth of beneficial probiotics like bifidobacteria, isobacteria, and clostridium IV in the intestinal tract [44,80]. Additionally, a positive association between *Bifidobacterium*-linked metabolic pathways and healthy urinary microbiota was noted [81,82]. However, these pathways cannot be inferred as protective in our dataset and should be interpreted as preliminary associations only. Of particular interest is the observation that certain viruses may act synergistically, leading to distinct alterations in the enteric bacteriome of immunocompromised individuals infected with these viruses [83].

Our study investigated the prevalence of HPyVs-JCPyV, BKPyV, and MCPyV in PC patients. JCPyV and BKPyV exhibited higher positivity rates and urine viral loads compared to tissues, consistent with their urinary tropism [84,85]. The presence of viral DNAs in urine may suggest a role in prostate carcinogenesis, particularly given that the intraprostatic reflux of infected urine could provoke inflammation, a recognized risk factor for PC [19,24,84,85,86]. Detection in BPH subjects and higher isolation in non-lesional versus lesional samples weakens a direct correlation [84]. However, the absence of viral DNA in lesional samples might be explained by the hypothesized *hit-and-run* mechanism in which the virus contributes to oncogenic transformation but is no longer detectable in tumor tissues [19,24,84,85]. MCPyV was not highly detected, supporting its limited tropism and lack of involvement in PC [87]. Focusing on HPyVs co-infections, JCPyV and BKPyV were found as the most common combination, consistent with their widespread distribution among the population [88]. Our findings support the renal epithelium as a preferential infection site for JCPyV and BKPyV, rather than MCPyV [87]. Moreover, the isolation of JCPyV and BKPyV in prostatic tissues confirms these viruses as common inhabitants of the prostate and suggests they may represent components of the prostate virome rather than direct oncogenic drivers. Since HPyVs-positivity alone is insufficient to establish a direct link to tumorigenesis, further research is needed to determine whether they are part of the normal prostate virome or contribute to carcinogenesis. This study has several limitations that should be carefully acknowledged. First, the sample size was limited, and no formal power calculation was performed; the study was conceived as an exploratory, pilot investigation, and the number of participants was determined by feasibility within the recruitment period.

Second, prostate tissue is intrinsically low-biomass matrix, increasing susceptibility to environmental or reagent-derived contamination. In this context, the predominance of Pseudomonas should be interpreted cautiously, as this genus is well-recognized contaminant in low-biomass microbiome studies.

Third, dedicated extraction blanks or reagent-only negative controls were not included in the sequencing workflow, limiting the ability to distinguish true prostatic taxa from potential background contaminants. This constraint may partly explain the absence of significant differences between lesional and non-lesional samples.

Fourth, correlations between urinary bacterial genera and volatile metabolites were modest in strength (Kendall τ ≈ 0.3–0.5). These associations should therefore be viewed as preliminary, hypothesis-generating signals rather than evidence of validated biological or clinical relationships.

Finally, the cross-sectional design prevents causal inference, and the multi-omic integration—although informative—should be confirmed in larger, independently replicated cohorts using optimized microbiological controls and high-resolution sequencing before any diagnostic implications can be considered.

## 5. Conclusions

In-depth research on the microbial community in PC, its intricate ecological interactions, and its influence on the prostate microenvironment is essential to advance prevention, diagnosis, treatment, and patient outcomes. Although no significant differences were observed between lesional and non-lesional tissues in this study, the consistent dominance of *Pseudomonas* highlights the challenges inherent to low-biomass prostatic samples and underscores the need for cautious interpretation.

In contrast, urinary samples showed clearer group-level differences, with several bacterial genera enriched in PC and associated with specific volatile metabolites. These observations, although not indicative of validated biomarkers, are preliminary and hypothesis-generating, suggesting that urinary microbiota and volatilome may reflect disease-related microbial–metabolic shifts.

Taken together, our findings support the concept that prostate carcinogenesis may involve complex interactions among host factors, microorganisms, and metabolic pathways; however, causality cannot be inferred. Larger, independently replicated studies are required to validate these exploratory signals and determine their potential clinical applications.

## Figures and Tables

**Table 1:** Clinical and pathological characteristics of the study population. (A) Clinical comparison between patients with histologically confirmed PC (*n* = 21) and individuals with BPH (*n* = 17). Continuous variables are expressed as mean ± standard deviation (SD), with median and range when appropriate. Categorical variables are reported as counts and percentages. Statistical comparisons were performed using Mann–Whitney U test for continuous variables and the chi-square test for categorical variables. (B) Pathological characteristics of the prostate cancer group, including EAU risk classification, tumor staging, ISUP grade, surgical margins, and biochemical recurrence. PC: Prostate Cancer; BPH: Benign Prostatic Hyperplasia; OSAS: Obstructive Sleep Apnea Syndrome; COPD: Chronic Obstructive Pulmonary Disease; PSA: Prostate-Specific Antigen; EAU: European Association of Urology; ISUP: International Society of Urological Pathology; R0: negative surgical margins; R1: positive surgical margins. * Statistically significant (*p* < 0.05).

A. Clinical Comparison Between PC Patients and BPH Controls			
Characteristic	PC Group (*n* = 21)	BPH Group (*n* = 17)	*p*-Value
**Age (years)**	65.50 ± 5.98 (67.0; 55–73)	64.70 ± 4.25 (64.0; 55–72)	0.6334
**Weight (kg)**	81.70 ± 16.18 (80.0; 65–110)	84.30 ± 12.35 (82.0; 75–110)	0.5779
**Height (m)**	1.74 ± 0.06 (1.73; 1.65–1.86)	1.76 ± 0.05 (1.74; 1.68–1.85)	0.2699
**BMI (kg/m^2^)**	26.90 ± 4.56 (26.64; 21.95–40.47)	26.30 ± 3.84 (26.40; 21.45–40.25)	0.6624
**Smoking status**	Yes: 5 (23.8%) No: 12 (57.1%) Ex-smoker: 4 (19.1%)	Yes: 4 (23.5%) No: 10 (58.8%) Ex-smoker: 3 (17.6%)	0.9926
**Family history of PC**	Yes: 3 (14.3%) No: 18 (85.7%)	Yes: 2 (11.8%) No: 15 (88.2%)	0.8192
**Family history of other cancers**	Yes: 10 (47.6%) No: 11 (52.4%)	Yes: 8 (47.0%) No: 9 (53.0%)	0.9726
**Comorbidities**	Hypertension: 16 (76.2%) Dyslipidemia: 5 (23.8%) Diabetes mellitus II: 1 (4.8%) OSAS: 1 (4.8%) COPD: 1 (4.8%) Allergic asthma: 2 (9.5%)	Hypertension:15 (88.2%) Dyslipidemia: 4 (23.5%) Diabetes mellitus II: 4 (23.5%) OSAS: 0 (0%) COPD: 0 (0%) Allergic asthma: 0 (0%)	0.3409 0.9839 0.0888 0.3619 0.3619 0.1911
**Prostate volume (cc)**	52.23 ± 24.20 (51.0;: 18.4–113.0)	77.42 ± 21.35 (80.0; 55.0–120.0)	0.0016 *
**Total PSA (ng/mL)**	8.10 ± 4.32 (6.80; 3.8–17.0)	4.24 ± 3.12 (4.80; 2.70–8.0.)	0.0029 *
**B. Pathological characteristics of PC group**			
**Characteristic**		**Distribution (n = 21)**	
**EAU Risk Classification**		Low: 3 (14.3%) Intermediate: 17 (80.9%) High: 1 (4.8%)	
**Pathologic stage**		pT2: 12 (57.1%) pT3a: 8 (38.1%) pT3b: 1 (4.8%)	
**ISUP Grade (at surgery)**		Grade 1: 3 (14.3%) Grade 2: 14 (66.7%) Grade 3: 4 (19.0%) Grade 4: 0 (0%)	
**Surgical margins**		Negative (R0): 14 (66.7%) Positive (R1): 7 (33.3%)	
**Biochemical progression**		No: 18 (85.7%)—PSA: 0.03 ± 0.01 (0.04; 0.01–0.05) Yes: 3 (14.3%)—PSA: 0.47 ± 0.12 (0.5; 0.4–0.6)	

**Table 2:** HPyVs median viral load in urine, non-lesional and lesional samples in prostate cancer patients. n: number of patients.

	Urine		Non-Lesional Samples		Lesional Samples	
	n (%)	Viral Load (Copies/mL), Median (CI 95%)	n (%)	Viral Load (Copies/mL), Median (CI 95%)	n (%)	Viral Load (Copies/mL), Median (CI 95%)
**JCPyV**	13/21 (61.9%)	8.7 × 10^6^ (2.25 × 10^5^–1.5 × 10^6^)	4/21 (19%)	1.38 × 10^3^ (1.4 × 10^2^–3.5 × 10^3^)	2/21 (9.5%)	4.65 × 10^2^ (2.5 × 10^2^–6.8 × 10^2^)
**BKPyV**	12/21 (57.1%)	1.33 × 10^5^ (3.2 × 10^4^–2.4 × 10^5^)	7/21 (33.3%)	1.2 × 10^2^ (9 × 10–2 × 10^2^)	2/21 (9.5%)	8.5 × 10 (8 × 10–9 × 10)
**MCPyV**	-	-	1/21 (4.8%)	1 × 10^2^	2/21 (9.5%)	1.16 × 10^2^ (1.1 × 10^2^–1.2 × 10^2^)

**Table 3:** List of VOMs significantly discriminating between the PC and the control group.

Metabolite Tag	IUPAC or Common Name	Class	Enrichment in PC Patients ^a^	*p*-Value
1	2,6-diisopropylphenol	Alcohol	↑	<0.0001
4	methyl-salicylate	Other	↑	<0.0001
27	octanal	Aldehyde	↓	<0.0001
34	p-cymene	Terpene	↓	<0.0001
31	decanal	Aldehyde	↓	0.0003
22	camphor	c-Ketone	↓	0.0004
9	nonanal	Aldehyde	↓	0.001
10	2,4.bis(1,1-dimethylethyl)phenol	Alcohol	↓	0.001
6	4-heptanone	Ketone	↓	0.002
21	theaspirane	Tetrahydrofurane	↓	0.002
18	2-ethyl-hexanol	Alcohol	↓	0.012
33	dimethyl-trisulfide	Sulfide	↓	0.031
20	tetradecane	Alkane	↓	0.034
39	pentadecane	Alkane	↓	0.037

**^a^** The symbols ↑ and ↓ denote higher and lower concentration, respectively, of the specified metabolite in PC patients compared to the control group.

## Data Availability

The raw data supporting the conclusions of this article will be made available by the authors on request.

## Abbreviations

The following abbreviations are used in this manuscript:

BPH	benign prostatic hyperplasia
VOMs	volatile organic metabolites
PC	Prostate cancer
HPV	*Human Papillomavirus*
EBV	*Epstein–Barr virus*
HHVs	human herpesviruses
CMV	*Cytomegalovirus*
KSHV	*Kaposi’s sarcoma-associated herpesvirus*
HPyVs	Human Polyomaviruses
MCPyV	Merkel cell polyomavirus
JCPyV	*JC* polyomavirus
BKPyV	*BK* polyomavirus
RP	radical prostatectomy
ST	sexually transmitted
PSA	prostate-specific antigen
mpMRI	multiparametric magnetic resonance imaging
EAU	European Association of Urology
PET-CT	positron emission tomography-computed tomography
RP	Robotic-assisted laparoscopic
ISUP	International Society of Urological Pathology
PSMA	Prostate-Specific Membrane Antigen
qPCR	Quantitative polymerase chain reaction
LTAg	Large T Antigen
VP1	Viral Protein 1
sTAg	small T antigen
GC	gas chromatograph/ chromatography
DAA	Differential abundance analysis
EAU	risk classification, tumor staging
ISUP	grade, surgical margins, and biochemical recurrence
OSAS	Obstructive Sleep Apnea Syndrome
COPD	Chronic Obstructive Pulmonary Disease
